# Morphology of Peripheral Vitreoretinal Interface Abnormalities Imaged with Spectral Domain Optical Coherence Tomography

**DOI:** 10.1155/2019/3839168

**Published:** 2019-06-09

**Authors:** Rachel L. Chu, Nicole A. Pannullo, Christopher R. Adam, Mohammad R. Rafieetary, Eric J. Sigler

**Affiliations:** ^1^Stony Brook University School of Medicine, 101 Nicolls Road, Health Sciences Center, Level 4, Stony Brook, NY 11794, USA; ^2^School of Chemistry and Materials Science, Rochester Institute of Technology, 1 Lomb Memorial Drive, Rochester, NY 14623, USA; ^3^Division of Retina and Vitreous, Kresge Eye Institute, Wayne State University, 4717 St. Antoine Street, Detroit, MI 48201, USA; ^4^Department of Vitreoretinal Surgery, Charles Retina Institute, 1432 Kimbrough Road, Germantown, TN 38138, USA; ^5^Division of Retina and Vitreous, Ophthalmic Consultants of Long Island, 2000 North Village Avenue, Suite 402, Rockville Center, NY 11570, USA

## Abstract

The objective of this study is to describe the clinical utility and morphologic characteristics of peripheral vitreoretinal interface abnormalities with spectral domain optical coherence tomography (SD-OCT). A prospective imaging analysis of 43 patients with peripheral vitreoretinal interface abnormalities seen on binocular indirect examination with scleral indentation was done. SD-OCT was evaluated for image quality and structural findings. Laser retinopexy was performed to surround all retinal breaks containing a full-thickness component via SD-OCT. Acceptable image quality for inclusion was obtained in 39/43 (91%) patients. Mean age was 41 ± 22 years, and mean follow-up was 14 ± 1.6 months. Decision to treat was altered following SD-OCT in 5% of the patients. Two cases of previously diagnosed operculated holes were found on SD-OCT to be partial-thickness operculated breaks or focal operculated schisis. Peripheral SD-OCT is a reliable and useful technique to examine the structural features of vitreoretinal interface abnormalities *in vivo*. This imaging modality is useful in the clinical management of suspected retinal breaks identified with indirect ophthalmoscopy.

## 1. Introduction

Peripheral vitreoretinal interface abnormalities span a range of entities from incidental ophthalmoscopic findings to retinal detachment. Findings such as lattice degeneration, white without pressure, vitreoretinal traction, and posterior vitreous detachment- (PVD-) associated retinal breaks are common reasons for the need to treat with retinopexy in order to prevent retinal detachment [1, 2]. Asymptomatic retinal breaks may lead to chronic inferior retinal detachment [3] and may occasionally be difficult to distinguish from retinoschisis [4]. Most of the current approaches to clinical management are based on indirect ophthalmoscopic interpretation [1–6]. Documentation of peripheral retinal pathology is typically limited to indirect ophthalmoscopy and fundus photography. More recently, wide-field fundus photography has become available and may be useful in detecting peripheral retinal pathology [7].

Similar to how spectral domain optical coherence tomography (SD-OCT) has advanced the interpretation of posterior pole pathology, SD-OCT may be a valuable method for evaluating peripheral vitreoretinal interface abnormalities [4, 8–13]. Most of the current knowledge about these peripheral entities is based on biomicroscopy [2], histopathological examination, and electron microscopy [14]. These modalities may not fully capture the accurate structural relationship between vitreous and retina *in vivo*. Recently, some authors have reported SD-OCT imaging findings with SD-OCT of lattice degeneration [9, 10, 15], white without pressure [11], retinoschisis [4, 16–18], and normative data for peripheral retinal thickness [19]. The authors recently observed retinal structural features on peripheral SD-OCT that were useful for clinical management and revealed some unexpected cross-sectional findings. The purpose of the present study was to prospectively evaluate the feasibility and clinical utility of peripheral SD-OCT of peripheral vitreoretinal lesions and examine their structural morphology *in vivo*.

## 2. Materials and Methods

This prospective, consecutive, and observational case series conformed to the tenets set forth in the Declaration of Helsinki and was performed in accordance with the Health Insurance Portability and Accountability Act of 1996. Consecutive patients presenting to a single vitreoretinal referral practice (Charles Retina Institute, Memphis, Tennessee) over a three-month initial study period with peripheral vitreoretinal pathology were included. All patients completed informed consent for imaging and study participation. All patients were examined with dilated slit lamp biomicroscopy and peripheral indirect ophthalmoscopy by a single experienced retina specialist including 360-degree scleral indentation. Color peripheral photography and SD-OCT (Spectralis, Heidelberg Engineering, Heidelberg, Germany) were performed through pathology identified on clinical examination. Patients with significant media opacity precluding a clear view of the peripheral retina and patients with peripheral retinal vascular disease were excluded. All patients were followed with repeat examination at three-month intervals if no treatment was indicated. Treated patients were followed at one-week, at one-month, and then at three-month intervals. No retreatment was indicated in the present series. All patients diagnosed with full-thickness retinal breaks were treated with focal laser retinopexy by author Eric J. Sigler (EJS) to completely surround retinal breaks using laser indirect ophthalmoscopy. Patients with less than 12 months of follow-up examinations were excluded.

### 2.1. Imaging

All images were obtained by a single, experienced ophthalmic photographer. Using a retinal drawing prepared by the examining physician, the photographer positioned the patient initially as for a standard SD-OCT image acquisition. The patient was then instructed to direct their gaze in the direction of the peripheral retinal lesion of interest. The lesion of interest was identified on the preimage scanning laser ophthalmoscopy (SLO) image, and the single line raster was positioned in a radial orientation through the lesion of interest. The image was then acquired using 25 B-scans/A-scan and displayed as a grey-scale B-scan. At least three parallel raster scans were obtained for each lesion. The images were then reviewed sequentially for morphologic features by two authors, EJS and Mohammad R. Rafieetary (MRR). Sufficient image quality was defined as the ability to visualize B-scan through the entire extent of the raster length and the presence of clear detail of retinal layers in at least three adjacent raster lines.

## 3. Results

Forty-three patients presented with peripheral SD-OCT findings. Acceptable image quality was obtained in 39 patients (91%). Poor image quality was due to media opacity in two patients and insufficient dilation in two patients. Mean patient age was 41 ± 22 years and consisted of 26 females and 17 males. Patient ethnicity included 26 Caucasians, nine African Americans, and four Hispanics. Mean follow-up was 14 ± 1.6 months. Peripheral retinal findings were as follows: white without pressure (WsP) (*n* = 8), lattice degeneration (*n* = 16), retinal break (*n* = 13), horseshoe break (*n* = 6), operculated break (*n* = 4), round atrophic break (*n* = 6), cystic retinal tuft (*n* = 6), degenerative retinoschisis (*n* = 3), and peripheral vitreoretinal traction (*n* = 2).

**Figure 1:**
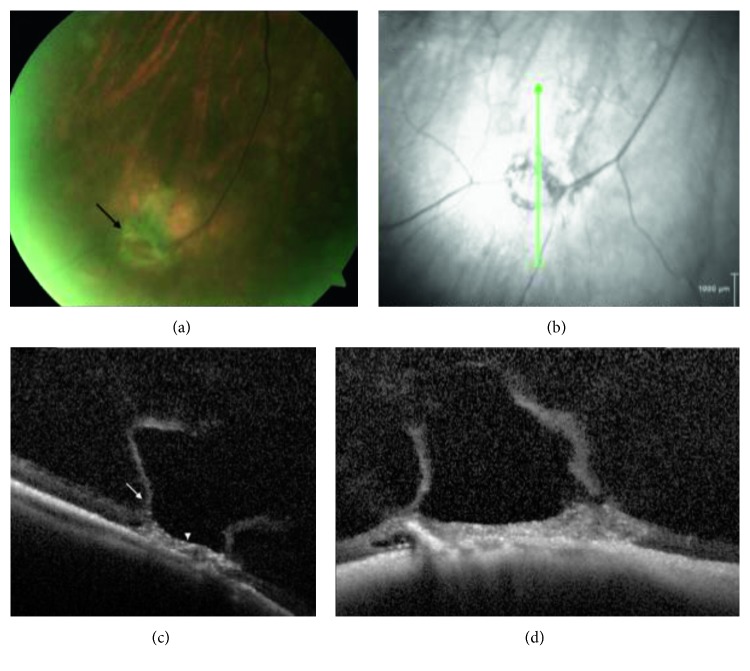
Peripheral SD-OCT of lattice degeneration with vitreoretinal traction. (a) Fundus photograph of the right eye with focal lattice degeneration and bridging vessel diagnosed with probable full-thickness break with ophthalmoscopy. (b) Scanning laser ophthalmoscopic image demonstrates circumferential hypoautofluorescence and scan position (green arrow) for SD-OCT in (c) and (d), which show the boundaries of cortical vitreous lacuna (arrow) overlying an area of lattice degeneration (arrowhead) with retinal atrophy, intraretinal pigment migration, and retinal pigment epithelium (RPE) irregularity; no full-thickness component was observed.

**Figure 2:**
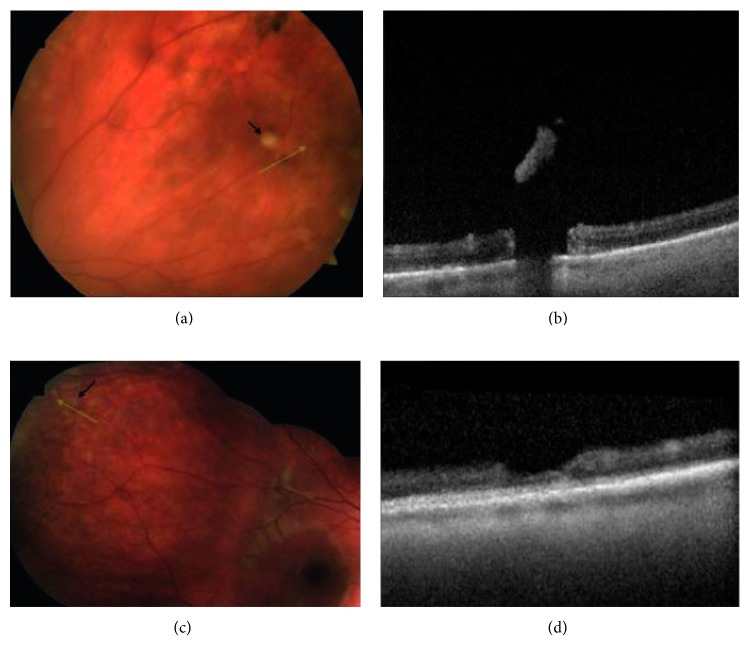
Peripheral SD-OCT of operculated breaks and focal operculated schisis. (a) Peripheral photograph of the left eye of this asymptomatic patient with an operculated break; black arrow denotes operculum; green line scan demonstrates scan position through the break. (b) SD-OCT reveals a full-thickness retinal break. (c) Photograph of the right eye of this symptomatic patient with an apparent full-thickness operculated break. (d) SD-OCT reveals focal operculated schisis with no full-thickness component.

**Figure 3:**
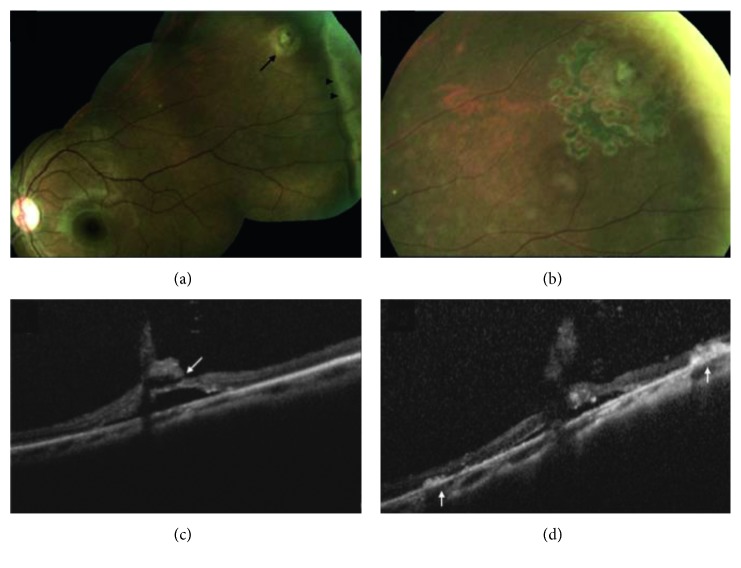
Peripheral SD-OCT before and after laser retinopexy for cystic retinal tuft with retinal break. (a) Fundus photograph of the left eye demonstrating cystic retinal tuft with retinal break (arrow) and white without pressure (arrowheads). (b) Appearance one month following laser retinopexy, demonstrating pigment corresponding to laser applications. (c) SD-OCT of the lesion before treatment, demonstrating full-thickness break with the tuft (arrow). (d) SD-OCT one week following laser treatment reveals outer retinal hyper-reflectance and early retinal pigment epithelium (RPE) pigment migration (arrows) corresponding to laser applications.

Lattice degeneration was present concurrently in six of the patients with WsP. Lattice degeneration with atrophic breaks was present in 9/13 patients with retinal breaks (Figure 1). In three patients, full-thickness retinal breaks were found present on SD-OCT that were not clinically detectable with ophthalmoscopy and scleral depression. Two cases diagnosed as vitreoretinal traction without retinal breaks on ophthalmoscopy were found to have a full-thickness component with SD-OCT. One patient diagnosed with having a full-thickness horseshoe break was found to have no full-thickness component with peripheral SD-OCT. Two out of four patients diagnosed with operculated retinal breaks with ophthalmoscopy were found to have a partial-thickness operculated break or focal operculated schisis (FOS) (Figure 2). No retinal detachments occurred within the study follow-up period. Patients treated with laser retinopexy revealed hyperreflective spots corresponding to the laser retinopexy (Figure 3) evident on both scanning laser ophthalmoscopic image and at the level of the outer retina by one week following therapy.

## 4. Discussion

The present series indicates that SD-OCT may be used to demonstrate peripheral vitreoretinal pathology and image some details that are not apparent with ophthalmoscopy. In eight patients (5%), the decision to treat or observe was changed following peripheral imaging. This is consistent with one previous report, in which OCT proved helpful in clinical decision-making when looking for the presence of subretinal fluid or elevation associated with lesions, uncovering subclinical retinal detachments and one presumed case of choroidal metastasis [9]. Additionally, SD-OCT was used to visualize the cross-sectional anatomy of laser retinopexy following treatment. We suggest that SD-OCT may be used to evaluate early chorioretinal adhesions in the period when acute white laser spots have faded and prior to the appearance of pigmentation.

A previous study of various peripheral retinal lesions using both time domain OCT (TD-OCT) and SD-OCT elucidated several findings [9]. The authors found that pigmented lattice degeneration tended to have thinner retinal layers and clinically unrecognizable breaks when compared to nonpigmented lattice degeneration. The present series did not demonstrate features common to idiopathic macular holes, such as circumferential subretinal fluid, focal opercula, hyaloid separation, or symmetric vitreoretinal traction surrounding atrophic holes. Another study evaluating lattice degeneration demonstrated the SD-OCT findings of previously histologically described structural elements [19]. These findings were consistent with observations in the present series, with cortical vitreous lacunae and retinal atrophy, with and without atrophic retinal breaks. WsP, which we [11] have recently termed “outer retinal whitening” due to SD-OCT findings, was frequently present surrounding and adjacent to lattice degeneration.

The present series identified the presence of partial-thickness operculated breaks, which we have termed “focal operculated schisis.” This is in apparent contrast to previous ophthalmoscopic definitions of operculated breaks [1–6]. Operculated retinal breaks have less commonly been associated with retinal detachment when viewed via ophthalmoscopy alone; this difference has been theorized due to the absence of remaining vitreoretinal traction in operculated breaks [3]. However, retinal detachment due to operculated breaks does occasionally occur [20]. The authors hypothesize that this difference is also due to the presence of partial-thickness retinal breaks, or FOS, in some cases that do not result in retinal detachment. Therefore, SD-OCT can be used to detect the presence of a full-thickness retinal break and the need for treatment or observation in the case of operculated schisis.

The present study is limited by its single-center design, the use of only one, high-quality imaging device, and a relatively short study period.

## 5. Conclusions

We conclude that SD-OCT is a useful tool in evaluating peripheral retinal pathology and that it reliably provides structural details that may change clinical management. Additionally, the presence of focal operculated schisis underlies a number of presumed operculated retinal breaks.

## Data Availability

The data used to support the findings of this study are included within the article.
